# The role of meningeal populations of type II innate lymphoid cells in modulating neuroinflammation in neurodegenerative diseases

**DOI:** 10.1038/s12276-021-00660-5

**Published:** 2021-09-06

**Authors:** Sherry Sin-Hang Yeung, Yuen-Shan Ho, Raymond Chuen-Chung Chang

**Affiliations:** 1 Laboratory of Neurodegenerative Diseases, School of Biomedical Sciences, LKS Faculty of Medicine, The University of Hong Kong, Pokfulam, Hong Kong SAR China; 2 School of Nursing, The Hong Kong Polytechnic University, Hung Hom, Hong Kong SAR China; 3 State Key Laboratory of Brain and Cognitive Sciences, The University of Hong Kong, Pokfulam, Hong Kong SAR China

**Keywords:** Neuroimmunology, Neuroimmunology

## Abstract

Recent research into meningeal lymphatics has revealed a never-before appreciated role of type II innate lymphoid cells (ILC2s) in modulating neuroinflammation in the central nervous system (CNS). To date, the role of ILC2-mediated inflammation in the periphery has been well studied. However, the exact distribution of ILC2s in the CNS and therefore their putative role in modulating neuroinflammation in neurodegenerative diseases such as Alzheimer’s disease (AD), multiple sclerosis (MS), Parkinson’s disease (PD), and major depressive disorder (MDD) remain highly elusive. Here, we review the current evidence of ILC2-mediated modulation of neuroinflammatory cues (i.e., IL-33, IL-25, IL-5, IL-13, IL-10, TNFα, and CXCL16-CXCR6) within the CNS, highlight the distribution of ILC2s in both the periphery and CNS, and discuss some challenges associated with cell type-specific targeting that are important for therapeutics. A comprehensive understanding of the roles of ILC2s in mediating and responding to inflammatory cues may provide valuable insight into potential therapeutic strategies for many dementia-related disorders.

## Neuroinflammation: Key role for lymphatic cells

Certain cells in the meningeal membranes around the brain appear to mediate inflammation of the CNS during neurodegenerative diseases. Type-II innate lymphoid cells (ILC2) reside in the lymphatic vessels in these membranes, which are increasingly recognized as important immune sites. Raymond Chuen-Chung Chang at the University of Hong Kong and co-workers reviewed studies of the distribution and activity of ILC2 in the CNS and its surroundings. They highlight promising evidence that ILC2 can modulate inflammatory triggers. There is still limited understanding of how ILC2 populations respond to damage within the brain, and limited methods for isolating and manipulating ILC2 populations. Future studies should investigate how ILC2 cells interact with inflammatory proteins, and how they could be exploited to treat brain inflammation, a common factor in neurodegenerative diseases including Alzheimer’s, Parkinson’s, and MS.

## Introduction

Neurodegenerative diseases describe a class of disorders that involve the progressive loss of function or structure within the central nervous system (CNS). Clinically, neurodegeneration may manifest in various ways, such as cognitive decline associated with progressive memory loss, motor degeneration, or a complex combination of both. Many neurodegenerative diseases, including Alzheimer’s disease (AD), multiple sclerosis (MS), and Parkinson’s disease (PD), have since evolved to further encapsulate psychiatric disorders, such as major depressive disorder (MDD). Early investigations into the pathogenesis of these neurodegenerative diseases revealed the involvement of key disease mechanisms, such as the upregulation of reactive oxygen species (ROS), reduced mitochondrial competence, changes in neural crosstalk, and the aggregation of toxic proteins, such as β-amyloid, tau, α-synuclein, and TDP-43, which is perhaps the most well-known mechanism.

**Fig. 1:**
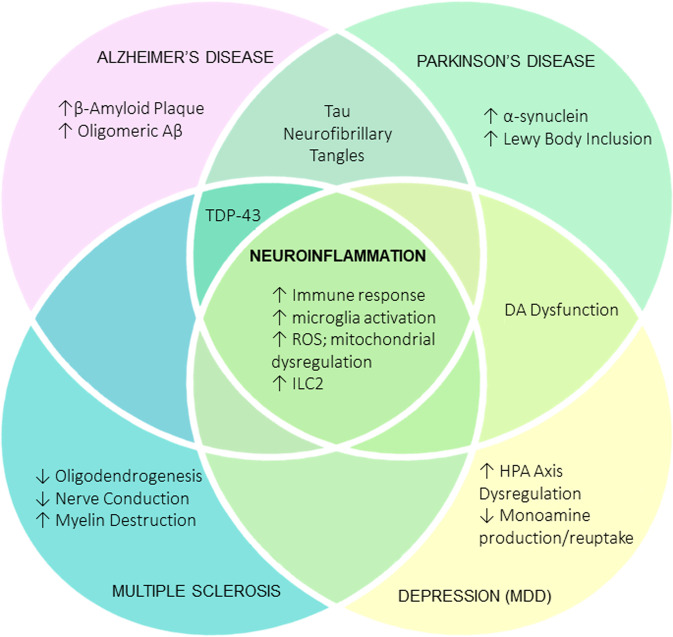
Schematic diagram illustrating the cellular damage that occurs in different neurodegenerative disorders, such as Alzheimer’s disease, Parkinson’s disease, multiple sclerosis, and depression (MDD). Although these neurodegenerative disorders share differences in pathology, they are connected by the upregulation of neuroinflammation (middle panel). Neuroinflammation is driven by an increased immune response, microglial activation, ILC2 activation, ROS, and mitochondrial dysregulation.

For obvious reasons, the pathophysiology of neurodegenerative disease is more complex than described here, in part due to the interactive and unpredictable nature of pathogenic proteins and a lack of understanding on how elements within these neurodegenerative diseases propagate functional and structural losses in the CNS. Clinical representations of these neurodegenerative diseases appear dissimilar upon initial scrutiny, such as the targeted loss of myelin in MS compared to more localized neuronal damage associated with the AD brain. However, recent evidence demonstrates that neuroinflammation is a common driving pathological mechanism in neurodegeneration due to its modulatory effects on common pathological proteins such as β-amyloid (Aβ) and tau (Fig. 1).

Numerous studies have reported that AD, MS, PD, and MDD exhibit rapid recruitment of inflammatory cues upon initial insult. More interestingly, the pathogenic brain also maintains a chronically elevated state of inflammation throughout disease progression^1–4^. In fact, the term “inflammation”, especially in the context of neurodegenerative disease, has achieved new importance in disease pathogenesis.

### Neuroinflammation in AD

Inflammation in AD has been investigated in basic and clinical research. The idea that conventional nonsteroidal anti-inflammatory drugs (NSAIDs) may delay cognitive decline and the pathological progression of AD is widely known^5,6^. In numerous animal studies, immune-related pathways, such as the complement pathway (i.e., C1q and C3) has been shown to be activated by the presence of Aβ^7,8^ and, more recently, the presence of tau^9^. Furthermore, pro-inflammatory cytokines such as IL-1, IL-6, IL-8, IL-34, and TNFα are upregulated in both mouse and human AD brains^10,11^. Closer analysis revealed that an increase in IL-1 dysregulates not only neurons but also astrocytes and microglia^12–14^, suggesting that inflammation can cause widespread damage to all cell types within the brain.

### Neuroinflammation in MS

Early studies of MS pathology demonstrated a strong correlation between inflammation and the extent of axonal injury. Of interest, translocator proteins identified in PET studies indicated increased innate immune activation in patients with secondary progressive MS compared to age-matched healthy controls^15,16^. Activated macrophages and T- and B-lymphocytes infiltrate the brain, where pro-inflammatory mediators and chemokines upregulate and activate brain-resident microglia^17,18^. This finding demonstrates that peripheral inflammation and subsequent demyelination in the dorsal root ganglion may contribute to MS-associated nerve lesions in patients. Hence, inflammation is an evident modulator of neurodegenerative diseases.

### Neuroinflammation in PD

In other neurodegenerative diseases, such as PD, longitudinal clinical studies have demonstrated that patients who regularly use anti-inflammatory drugs, such as ibuprofen, had a later disease onset^19^. It became important to temporally determine whether inflammation acted as a trigger of pathology or vice versa. Triggering brain inflammation via the activation of TLR3 in the SNc of adult rats resulted in cytoplasmic mislocalization of TDP-43^20^. This mislocation was associated with the susceptibility of DA neurons to 6-OHDA, a neurotoxic trigger. More interestingly, systemic antagonism of IL-1R attenuated inflammatory stress and TDP-43 pathology within these same DA neurons. These results collectively indicate that inflammation is a vital regulator of PD pathology. Other studies have also suggested that the activation of immune cells such as natural killer (NK) cells can modulate neuroinflammation induced by α-synuclein through interactions with microglia. In fact, the depletion of NK cells can exacerbate synucleinopathies through decreased surveillance^21^. Although neuroinflammation has been shown to exacerbate pathologies, the activation of immune cells in PD may be more complex than previously appreciated.

### Neuroinflammation in MDD

Similarly, DA neuronal damage is not exclusive to PD but is also observed in MDD (depression). Studies investigating inflammatory cues in depression have suggested that inflammatory cytokines affect DA neurons in the ventral striatum to produce robust symptoms related to motivation^22^. Neuroendocrine studies have also demonstrated increased HPA axis modulation associated with higher levels of cortisol release^23^. Overactivity of the hypothalamus in the HPA axis, as well as excess activation of the amygdala, promotes the recruitment of macrophages^24^ and a surge in cytokine release. Interestingly, pro-inflammatory cytokines have also been shown to deplete monoamine neurotransmission and reduce neurotrophic factor release, leading to irreversible glial damage and acute neuronal apoptosis. Collectively, the importance of neuroinflammation in the pathogenesis of neurodegeneration cannot be denied and warrants further investigation.

## Immune Crosstalk Between the Brain and Periphery

Brain immunity was previously understood to be controlled in isolation by brain resident macrophages such as microglia. Activated microglia and astrocytes are hallmarks of pathology, and several compounds have been proposed to modulate their activation. Decades of research indicate that the role of microglial activation in disease is complex, as both beneficial and detrimental effects of microglial activation have been extensively described. For instance, microglial activation can release pro-inflammatory cytokines (e.g., TNFα), leading to reductions in cognition^25^. Conversely, treatment of microglia with IL-10 prevents pathological hyperactivation^26^. The relative contributions of local cytokines to the microglial response and how this is presented in complex disease states are still largely inconclusive. However, recent investigations have pointed out that peripheral populations of immune cells (e.g., peripheral macrophages) can also actively modulate neuroinflammation by entering the brain via either the BBB or meningeal lymphatic vessels (MLVs).

Early investigations into peripheral neural inflammatory crosstalk indicated that the BBB was a possible platform. Indeed, the BBB is a regulator of molecular exchange in and out of the brain parenchyma. Extensive experimental evidence has demonstrated the direct movement of cytokines through the BBB. For instance, TNFα in the vasculature moves directly across the BBB ~30 min postinjection^27^. Mechanisms by which neuroinflammatory molecules directly cross the BBB may include increased permeability in disease states^28^. Endothelial cells within the BBB have been shown to be compromised during neuroinflammation, leading to an uncontrolled and unfavorable influx of inflammatory cues.

Although BBB integrity has been shown to be compromised in neurodegenerative disease, few macrophages and cytokines are transported within the vasculature under normal conditions. The infiltration of inflammatory signals from the BBB only occurs when considerable damage has already been induced. Unlike the BBB, the meningeal space (e.g., CSF) already carries numerous surveillance immune cells under healthy conditions. Meningeal endothelial cells are more permissive than other cells due to a lack of astrocytic end-feed^29^. Tracing studies have demonstrated significant differences in draining properties between the meningeal and parenchymal compartments. For instance, tracers injected into the subarachnoid space reach the cervical lymph nodes first, demonstrating that CFS drainage can easily occur outside the CNS and propagate an immune response. Consistently, mouse models of MS demonstrate that myelin antigens accumulate first in the cervical lymph nodes^30^. Similarly, β-amyloid was also detected in cervical lymph nodes in AD mouse models^31^, and deep ligations resulted in aggregated pathology^32^. Collectively, the role of the meningeal space and meningeal lymphatics in supporting crosstalk between the periphery and brain environments cannot be ignored.

## Meningeal Lymphatics

As the meningeal compartment is proximal to the brain but lacks BBB innervation, it is more easily accessible by the periphery. These attributes allow the meningeal space to serve as an effective communication route between the immune cells in the periphery and CNS. Long thought to merely serve as buoyancy and protection for the CNS, the meninges and lymphatic drainage have increasingly been recognized to modulate both homeostatic and pathological brain functions.

Most notably, MLVs contain immune cells in circulating cerebral spinal fluid (CSF), even under healthy conditions^33,34^. Initial investigations into the immune role of meningeal lymphatics revealed the importance of meningeal T-lymphocyte populations in regulating cognition. More specifically, meningeal T-lymphocytes have been shown to produce IFNγ and IL-4, which have regulatory effects on social behavior and cognition^35,36^.

The involvement of MLVs in neurodegeneration has been demonstrated in both AD and PD. Increased accumulation of toxic protein aggregates such as β-amyloid^37^ and α-synuclein^38^ occurred as a result of drainage depletion within MLVs. As a proof of concept, localized injection of VEGF in a transgenic AD mouse model ameliorated the β-amyloid plaque burden and rescued cognitive deficits^39^. Furthermore, dysregulated meningeal lymphatic drainage resulted in decreased β-amyloid clearance by anti-AB immunotherapy^40^. In the experimental autoimmune encephalomyelitis (EAE) model of MS, the meningeal compartment revealed the early activation and recruitment of encephalitogenic T-cells within the lymphatics^41^, suggesting a major role of the meninges during early disease onset. Overall, this evidence suggests that meningeal compartments are extremely dynamic and modulate the activation of immune cells from the periphery to the CNS.

### ILC progenitors and origin

**Fig. 2:**
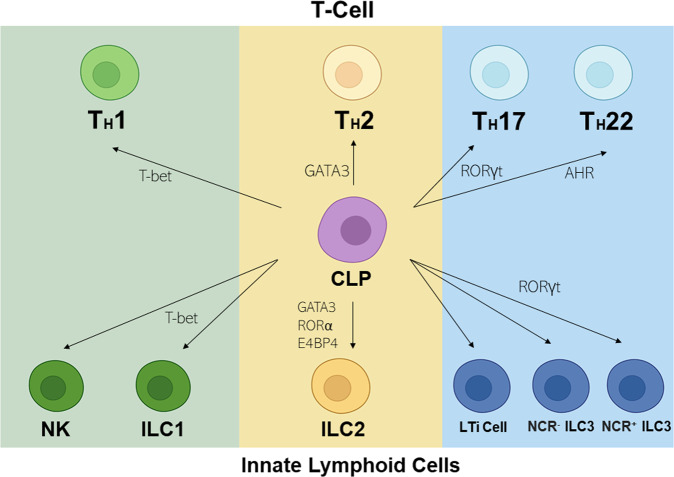
Schematic diagram summarizing the similarities and differences in transcription factor expression between T-cell and ILC subtypes (NK cells/ILC1s, ILC2s, ILC3s). T-bet promotes the differentiation of NK cells/ILC1s, while GATA3, RORα, and E4BP4 promote ILC2 differentiation, and RORγt promotes LTi cell, NCR^−^ ILC3, and NCR^+^ ILC3 differentiation. Illustration created in part with *BioRender.com*.

During the early stages of fetal development, ILCs function as lymphoid tissue-inducer cells (LTi cells)^42^. These cells induce the development of secondary lymphoid tissues by instructing mesenchymal stromal cells to produce and retain hematopoietic cells^43^. Although three primary groups of ILCs have been classically identified (i.e., ILC1s, ILC3s, and ILC2s), these cells present much higher plasticity in their lineage than previously assumed. The various branches of the ILC family share a common ancestry and developmental pathways. For instance, all ILCs require Notch signaling during development^44^. Furthermore, the reliance on ID2 and α4β7 integrin as common developmental progenitors indicates that ILCs might be derived from the same precursor (Fig. 2).

However, recent studies examining ILC lineage during development indicate that additional complexity and plasticity exist within this arm of hematopoiesis. The involvement of many transcription lineages suggests that the final fate of ILCs is highly malleable. For example, ILC3s are derived from both α4β7^−^ CLPs via Notch signaling and from LTi cells in the periphery. Moreover, proliferating ILC3s may lose RORγt expression in the absence of IL-7 signaling and give rise to INF-γ-producing ILC1s. This evidence demonstrates a level of complexity and plasticity in ILC development. These lineage behaviors should be further studied in the context of the brain and, more importantly, whether this behavior may change in response to the build-up of toxic proteins in neurodegenerative diseases.

### ILC1s in the meningeal lymphatic vasculature

Although research within the last 5 years has shed considerable light on the role of meningeal drainage in modulating neuroinflammation, many complex cell types within MLVs remain to be fully elucidated. For instance, meningeal populations of type I innate lymphoid cells (ILC1s) have been shown to promote the infiltration of T_H_17-mediated pro-inflammatory cytokines and chemokines directly into the parenchyma of the brain and spinal cord^45^. Furthermore, a comparison of ILC1s and NK cells revealed that the choroid plexus mainly contained ILC1 populations and that chemokines (i.e., CXCL16) can promote the infiltration of these cells into the brain parenchyma^46^. This evidence collectively suggests that ILC1s in the CNS act as distinct gatekeepers involved in the modulation of neuroinflammation in a model of EAE and may play important roles in propagating an initial neuroimmune response to early CNS insults.

### ILC3s in the meningeal lymphatic vasculature

Type III innate lymphoid cells (ILC3s) in the periphery are characterized by the expression of RORγt and can be subdivided into two transcriptionally and functionally heterogeneous groups in adults: LTi-like ILC3s and NCR^+^ ILC3s^47^. Within the CNS, RORγt^+^ ILC3s have been shown to populate the meninges. These same populations were increased in a model of EAE and promoted IL-17 production. Furthermore, ILC3 deficiency in mice reduced immune T-cell trafficking to the meninges in the context of EAE^48^, demonstrating an important role in T-cell maintenance within the CNS.

### ILC2s in the meningeal lymphatic vasculature

Type II innate lymphoid cells (ILC2s) were also recently shown to reside within MLVs, particularly within the CSF-producing choroid plexus and around the dural sinus. Recent investigations revealed a previously underappreciated role of ILC2s in modulating processes such as cognition and neuronal repair. Although ILC2s were first identified at barrier surfaces of cells in the periphery (e.g., lung), recent research has shown that these cells also highly populate the brain and spinal cord^49,50^. The identification of this unique cell type within the CNS has therefore inspired investigation into whether ILC2s can modulate neuroinflammatory cues during aging and neurodegenerative disorders, including their potential reparative properties after CNS insult.

### Possible interactions of ILCs within the meningeal lymphatic vasculature

**Fig. 3:**
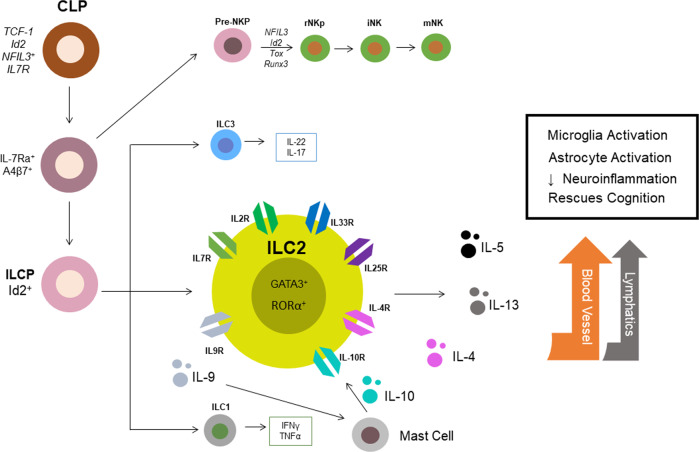
Development and downstream effectors of innate lymphoid cells, with particular attention given to ILC2s. In adults, ILCs initially differentiate from common lymphoid progenitors (CLPs), which are commonly found in the bone marrow, via notch signaling. Transcription factors promote the differentiation of CLPs into ILC precursors (ILCPs), which further differentiate into NK cells, ILC1s, ILC3s, and ILC2s. Of interest, ILC2s express many surface receptors (e.g., IL7R, IL2R, IL33R, IL25R, IL4, IL4R, IL10R, and IL9R). Cytokines (dots) such as IL-5 and IL-13 are robustly produced by ILC2 stimulation and may activate microglial populations through pathways such as blood vessels or lymphatic drainage. Ultimately, ILC2 activation in disease may induce microglial activation and astrocyte activation, repress neuroinflammation and ameliorate cognitive deficits in an aging model.

The contrasting effects of ILC1s and ILC3s in a model of traumatic brain injury (TBI) revealed that the activation of ILC2s through IL-33 simulation resulted in suppressed ILC1 and ILC3 populations within the meninges in both healthy and Rag1^−/−^ mice^51^. This finding demonstrates some levels of cross-modulatory effects between ILC subtypes, despite obvious etiological differences in their upstream transcriptional activation behavior (Fig. 3). Additionally, AMPK stimulation suppressed pro-inflammatory ILC1/3 populations, which may ameliorate the secondary neuronal death commonly observed in models of TBI. In AD models, AMPK activation was also shown to ameliorate both Aβ and tau pathologies. Although the effects of ILC1/3s generally seem to reduce pro-inflammatory insults in CNS diseases, it is important to independently investigate their effects on TBI and neurodegeneration. It is likely that the modulatory effects of ILC subtypes depend on the temporal nature of the insult, as TBI induction is rapid, while neurodegeneration is progressive in comparison. The effects of ILC1/3s on neurodegenerative models are less well understood than those of ILC2s. Additionally, the cross-modulatory effects of these different ILC subtypes within the brain are not well understood within the literature, and a deeper appreciation on the scale of their collective involvement in guarding brain immunity in both aging and neurodegeneration will be needed. As the role of ILC1s and ILC3s in the brain remains elusive and have only been described in the context of rapid brain injury, only ILC2s will be discussed in the context of neurodegeneration within this review.

## ILC2s and the Immune Responses in the Brain and Periphery

Compared to other ILC subtypes, type 2 ILCs (ILC2s) are the most well defined within the CNS. The results of a genome-wide transcriptional profiling study demonstrated that many neuron-specific genes were selectively enriched only in ILC2s compared to their counterparts (i.e., T-cells, NK cells/ILC1s, and ILC3s) (Table 1), suggesting that ILC2s are the main subtype expressed within the brain. ILC2s directly localize in the brain and robustly modulate neuroinflammation through interactions with downstream cytokines.

**Table 1:** Summary of the types of innate lymphoid cells (ILCs), including T_H_ cell types, transcription factors, cytokine involvement, and distribution within human peripheral and CNS tissues.

Characteristics	NK cell/ILC1		ILC3		ILC2	
**T-helper cell type**	T_H_-1		T_H_-1		T_H_-2	
**Transcription factors**	RORγt^−^, Gata3^+^, T-bet^+^ (ILC1), Eomes^−^ (ILC1), T-bet^−^ (NK), Eomes^+^ (NK)		RORγt^+^, Gata3^+^, T-bet^+^, Eomes^−^, Ahr^+^		, Gata3^+^, T-bet^−^, Eomes^−^	
**Cell surface markers**	CD45^+^, CD69^+^, CD117/c-kit^−^, IL-2Rα^+^, IL2Rβ^+^, CXCR3^+^, IL12Rβ2^+^, IL-17Rβ^−^		CCR6^+^, CD25/IL2Rα^low^, CD45^+^, CD4^−^, CD90/Thy1^+^, CD117/c-kit^+^, IL23R^+^		CD4^-^, CD45^+^, IL-2Rα^+^, CD90/Thy1^+^, CD161^+^, KLRG1^+^, ST2/IL33R^+^, TSLPR^+^	
**Activated by**	IL-12, IL-15, IL-18		IL-1β, IL-23		IL-33, IL-25, TSLP	
**Downstream cytokine**	IFNγ, TNFα, Perforin, Granzymes		IL-17, IL-22, GM-CSF		IL-5, IL-13, IL-4, AREG	
**Physiological purpose**	Macrophage activation cytotoxicity oxygen radical response		Macrophage activation phagocytosis antiviral/antimicrobial		Macrophage activation allergic reaction mucus production vasodilation extracellular tissue repair	
**Peripheral distribution** (Kim et al., 2016)	Bone marrow large intestine mesenteric lymph node		Small intestine large intestine peripheral lymph node		Lung small intestine skin adipose	
	Liver		Lung, spleen		Liver, bone marrow peripheral lymph node	
	Lung		Adipose		Large intestine	
**CNS Distribution**	**Health**	**Disease**	**Health**	**Disease**	**Health**	**Disease**
	CP^46^	Brain parenchyma^46^	Meninges^47^ CP^47^	Meninges^47^ CP^47^	Meninges^50^ CP^49^	Meninges^50^ CP^49^, leptomeninges^48^

Peripheral tissue distributions are categorized as high expression (orange), moderate expression (yellow), and low expression (off-white) for each ILC subtype. Additionally, the CNS distribution of each ILC subtype in health and disease is summarized.

Upon CNS injury, alarmins (e.g., IL-33) expressed by healthy glia activate ILC2s^52^. Subsequently, ILC2s promote the release of various cytokines in the interleukin family and further modulate inflammation. A model of spinal cord injury (SCI) indicated a surge of IL-33 within the cerebrospinal fluid (CSF), leading to the activation of ILC2s within the spinal cord meninges^49^. Interestingly, no ILC2s were detected in the meninges of the spinal cord in healthy counterparts, suggesting the ability of these cells to switch between functionally dormant and proliferative states in the CNS in response to IL-33 stimulation. Other studies have demonstrated that dormant ILC2s exist as ILC precursors within bone marrow^53^. However, it has not been confirmed whether the meningeal population of ILC2s shares the same activation profile as those found in the periphery. Peripheral ILC2s have been shown to differentiate into either IL-25R^+^/IL-33R^−^ inflammatory ILC2s (iILC2s) or IL-25R^+^/IL-33R^−^ natural ILC2s (nILC2s), primarily through stimulation with IL-25 or GATA-3, respectively^54^. It is currently unclear whether meningeal populations share such a complex progenitor differentiation fate compared to their peripheral counterparts. In the periphery, iILC2s are unresponsive to IL-33 stimulation, but most studies describing CNS populations of ILC2s demonstrate some levels of modulation by IL-33^50,52,55^, suggesting that perhaps iILC2 populations may be far fewer (if not nonexistent) than IL-33-activated nILC2s.

Prior to the recent discovery of ILC2 populations within the brain, this unique cell type was most extensively studied in mucosal tissues in the periphery, including the lung, small intestine, bone marrow, spleen, liver, kidney and adipose tissues^56,57^. ILC2s are important modulators of allergic inflammation and inhibit helminths, and unlike neural populations, these cells are continuously expressed within these tissues. Within the periphery, ILC2s have been shown to express CD90 and IL-7Rα^58,59^. Furthermore, their development and function rely on the transcription factors GATA3, RORα, ID2, and NFIL2^60^. Experimental evidence suggests that Tcf7 highly promotes ILC2 development in either a GATA3-dependent (through the upregulation of IL-17Rβ and IL-2Rα) or GATA3-independent manner (through the upregulation of IL-7Rα alone). Tcf7 mice deficient in TCF-1 demonstrate markedly reduced numbers of ILC2s in the lungs and bone marrow compared to their wild-type counterparts^61^. However, functional TCF-1 deficiencies do not completely eradicate all production of ILC2s. Several groups have proposed alternative proteins that modulate the production of ILC2s within the periphery (e.g., COX)^62,63^, but these mechanisms are still under heavy investigation.

In the brain, meningeal ILC2s have been shown to have similar surface marker profiles as their peripheral counterparts and express C-kit, CD25, and IL-7Rα^64^. A large proportion of RORγ^+^ cells were also found within the brain, indicating the presence of healthy proliferating ILC2 populations. Collectively, this evidence suggests that ILC2s in the brain and periphery may express similar proteins and transcription factors. However, their dormancy or proliferative states may differ. Granted, our understanding of neural populations of ILC2s is still limited but rapidly growing. Further investigations should seek to elucidate the complex mechanistic differences between ILC populations in the brain and periphery.

## Brain Surveillance by Meningeal-Specific Populations of ILC2s

Both CNS and peripheral populations of ILC2s share a large number of similarities in developmental and translational proteins. Although ILCs are mainly tissue-resident cells with organ-specific interactions, they have also been shown to reside within the brain parenchyma and in the meninges of the brain^49,50^. More importantly, both CNS and peripheral populations of ILC2s have been shown to be activated by a similar set of cytokines (i.e., IL-33 and IL-25) and produce downstream effects on a similar set of cytokines (i.e., IL-5, IL-13, and IL-10). As ILC2s are modulated by cytokines and simultaneously modulate downstream cytokines within the CNS and periphery, it would not be surprising to systemically activate peripheral populations of ILC2s to modulate CNS populations of ILC2s, and vice versa. A deeper understanding of this phenomenon is crucial, as the therapeutic potential of ILC2s depends on the nature and extent of the crosstalk between their systemic and neurological populations through meningeal lymphatics.

Although most ILC2 populations within the periphery are resident and relatively immobile, the downstream cytokines produced by ILC2s have been shown to be actively regulated between the brain and the periphery. Cytokines produced by meningeal immune cells can readily infiltrate the brain via the CSF^65,66^ and induce secondary activation of local glial-immune cells such as microglia. It would be unsurprising for ILC2 populations within the meninges to be activated by both brain and peripheral IL-33 and then proceed to release downstream cytokines that affect neural cells and their neuroinflammatory cascade. The following section will examine some of the basic and preclinical investigations on cytokines and chemokines that can modulate or are modulated by ILC2s (Table 2).

**Table 2:** Summary of some studies investigating the effects of cytokines that are downstream of ILC2s on neuroinflammation in the context of aging, Alzheimer’s disease, multiple sclerosis, Parkinson’s disease, and depression (MDD).

Neurodegenerative disorders	ILC-modulating cytokines	Basic/preclinical evidence	Reference	Human clinical evidence	Reference
Aging	IL-33	Downregulation of IL-33 resulted in the loss of neurons in the cerebral cortex and hippocampus and increases in tau abnormality in aged mice	^50,156^	Not directly investigated	-
	IL-5	Activation of IL-5 in aged mice increased the formation of new nerve cells in the hippocampus.	^50^ ^157^	IL-5 is decreased in aged/senescent human brains	^158^ ^159^
	IL-13/4	Exercise can upregulate IL-13/IL-4 concentrations and promote the expression of M2-associated genes in the hippocampus	^160^ ^161^	IL-13 is associated with senescence in humans in a cross-sectional blood collection study	^162^
	IL-10	IL-10 is associated with increased microglial activation and reduced inflammation in aged brain and the POCD model	^163^ ^164^	Human brain samples indicate that IL-10 is associated with inflammaging in the middle-aged community	^165^ ^166^
	CXCL16/CXCR6	CXCL16 increases mEPSC, modulating GABA release in CA1 hippocampal neurons	^94^	Serum CXCL16 levels are associated with age-related stroke incidence	^167^ ^168^
Alzheimer’s disease (AD)	IL-33	IL-33 ameliorates Alzheimer’s-like pathology through modulating Aβ and tau.	^55^ ^108^	Brains from human AD patients exhibit strong IL-33 expression in the vicinity of Aβ and NFTs	^169^ ^170^
	IL-5	Upregulation of IL-5 is neuroprotective in cell cultures and 3x Tg AD mice models	^50^ ^109^ ^110^	Postmortem AD brains show that IL-5 changes are associated with pathological severity	^77^
	IL-13/4	IL-13 and Il-4 can modulate AD pathology in cell cultures and mouse models	^149^ ^171^ ^172^	Levels of IL-13/IL-4 correlate with mild cognitive impairment in AD patients	^173^ ^174^
	IL-10	IL-10 deficiency is associated with improved AD outcomes in mouse models	^175^ ^176^	Serum IL-10 levels in the CSF of AD patient correlate with the amount of amyloid beta deposition	^177^ ^178^
	TNFα	TNFα is associated with increased Aβ plaques and tau tangle burdens	^105^ ^106^	AD brains exhibit increased TNFα. Anti TNFα drugs have been tested in human subjects	^104^ ^107^
Multiple sclerosis(MS)	IL-33	Activation of IL-33 via ILC2s removes susceptibility in a mouse model of EAE. Blockade of IL-33 removes protection against EAE insult. IL-33 also upregulates oligodendrocyte-mediated protection	^64^ ^114^ ^115^	Increased concentrations of IL-33 in the serum and CSF of MS patients	^179^
	IL-5	IL-5 supports a shift to Th2 immunity	^180^	IL-5 levels are associated with positive responses to Glatiramer acetate treatment in MS patients.	^181^
	IL-13/-4	IL-13/4 supports a shift to Th2 immunity	^180^	IL-13 levels are associated with positive responses to Natalizumab treatment in relapse-remitting MS patients.	^181^ ^182^
	IL-10	IL-10 activation by BBI administration leads to delayed onset of EAE	^183^ ^184^	Lower IL-10 expression is correlated with higher lesions in demyelinating diseases	^185^ ^186^
Parkinson’s disease(PD)	IL-33	IL-33 release in bone marrow-derived cultures in the presence of dopamine toxin	^187^	Not directly investigated	-
	IL-5	IL-5 upregulation via VIPs induces changes in the gut microbiota and decreases pathological burden	^130^	Not directly investigated	-
	IL-13/4	IL-13 may be neuroprotective by reducing inflammation via the death of pro-inflammatory microglia. Alternatively, IL-13 can also exacerbate neuronal death in PD models	^188^ ^150^ ^189^ ^190^	IL-13 is associated with cellular susceptibility to oxidative stress in idiopathic PD patients	^191^
	IL-10	Gene transfer of human IL-10 into a rat model of PD may be neuroprotective	^192,193^	Plasma levels of IL-10 are associated with PD severity and progression	^194^
Depression(MDD)	IL-33	IL-33 release is associated with microglia activation and worsens inflammation	^195^	IL-33 is associated with an increased risk of depression in women with a history of childhood abuse	^196^ ^197^
	IL-5	IL-5 upregulates the Ras-ERK pathway, which causes deficits in synaptic plasticity and motivation	^198^	Upregulated levels of serum IL-5 are associated with increased MDD in children	^199^ ^200^ ^201^
	IL-13/4	IL-4 and IL-13 enhances MAO-A expression, leading to the upregulation of serotonin metabolism	^202^	Depressed patients who are associated with obesity have higher levels of IL-13 than nondepressed patients	^203^
	IL-10	IL-10 administration rescues learning and memory deficits in a model of depression in mice.	^145^ ^204^	Low serum IL-10 is associated with the dysregulation of IL-6 in MDD patients	^205^

### IL-33

IL-33 is a potent activator of ILC2s in both the periphery and CNS. IL-33 belongs to the IL-1 cytokine family, which includes IL-1β and IL-18^67^. Unlike other members of the IL-1 family, IL-33 is expressed at high levels in glial immune cells within the CNS^68,69^.

Due to the wide array of effects of IL-33 in both the CNS and periphery, ongoing research is closely examining the effects of IL-33-induced ILC2 activation in the context of CNS insult. Previous studies have demonstrated that IL-33 activation is pro-inflammatory in nature and promotes the induction of epithelial cells and endothelial cells^68^. The activation of IL-33 specifically within mast cells in PD models induced further activation of astrocytes and high levels of p38 and NFκB, which are prominent signaling machinery for pro-inflammatory cytokines^70,71^. In contrast, a model of retinal detachment via Müller cell gliosis demonstrated that IL-33 deficiency could help ameliorate pathogenesis by reducing the recruitment of pro-inflammatory cytokines such as IL-1β, IL-6, and TNFα. In the context of AD, impairments in IL-33/ST2 signaling have been shown to be increased in patient serum. Treatment with IL-33 has been shown to induce synaptic plasticity and ameliorate cognitive deficits in PS1 mouse models^55^. The controversial effect of IL-33 activation on disease could be due to its effects on specific cell types (i.e., mast cell, endothelial cells, or glial cells). Indeed, IL-33 receptors are widely expressed on these cell types^63,69^. Therefore, the varying effects on pathology may not entirely be surprising.

In a model of PLP_139–151_-immunized SJL mice (MS attenuation), IL-33 was significantly reduced in multiple tissues^72^, suggesting that these cells are quiescent during nondisease states. The evidence clearly demonstrates that in disease, IL-33 triggers ST2 + ILC2s to produce IL-13 and other T_H_2-polarizing cytokines. Interestingly, when administered at the peak of clinical symptoms, IL-33 prevents relapse by inducing ILC2 activation in the meninges and CNS and the release of pro-inflammatory cytokines. It is understood that the release of these pro-inflammatory cytokines by IL-33-induced ILC2s ameliorates this damage^73^. Collectively, this evidence demonstrates that through potent activation by IL-33, ILC2s can alleviate symptoms in a model of EAE by modulating cytokines. The following sections will examine how these cytokines can modulate and attenuate neurodegenerative disorders.

Despite the promising interactions demonstrated between IL-33 and ILC2s, it remains important to note that IL-33 is pleotropic and modulates the activation of several other neural cell types. For instance, the loss of neuronal or microglial IL-33 receptors leads to impairments in spinal plasticity and reduced consolidation of fear memories. Clearly, IL-33 is vital for modulating synaptic plasticity and age-related decline in cognition^74^. Consistently, the administration of IL-33 to animals has also been demonstrated to increase cognitive function^75^. It is still unclear whether the cognitive improvements seen in these experiments are due to independent effects of microglia and ILC2s or a combination of their effects after activation. Further studies will elucidate the complex interrelationship between microglia and ILCs in response to IL-33 activation and their exact roles in modulating cognition in both healthy and disease states.

### IL-5

IL-5 is a multipotent cytokine that is produced primarily by ILC2s. Cytokines, such as IL-5, are signaling molecules within the immune system that affect the synthesis, release, and cell reuptake of monoamines. While many studies have reported that a majority of IL-5-producing cells are present in the lung and intestine, recent evidence suggests that ILC2s located within the meninges and choroid plexus produce a large portion of IL-5^49,50^. Perhaps unsurprisingly, many early studies also demonstrated that astrocytes and microglia produce IL-5. The proliferation and activation of microglia were induced by IL-5 simulation^76^. It remains likely that IL-5 release by ILC2s can modulate microglial recruitment to some extent. However, this phenomenon has not yet been directly documented within the literature and requires further examination.

IL-5 has been shown to promote neurogenesis in the hippocampus and reduce neuroinflammation^50^. An early study using PLSR analysis in AD patient samples identified IL-5 as one of three cytokines that most strongly correlated with pathological severity^77^. The induction of IL-5 by IL-33 has been shown to reduce atherosclerotic plaque formation^78^, although it is unclear whether this effect can be modulated by IL-5 produced specifically by ILC2s. In PD, IL-5, and GCSF levels correlated with both cognitive and motor dysfunctions^79^, demonstrating its dual roles. Further research is needed to elucidate whether the function of IL-5 in disease progression is dependent upon the ILC2-specific modulation of IL-5.

### IL-13

IL-13 can downregulate the synthesis of type-1 T helper (Th1) lymphocyte pro-inflammatory cytokines and is therefore anti-inflammatory in nature. Early studies indicated that microglia selectively express IL-13 and promote neuronal survival in ischemic models via a reduction in neuroinflammation^80^. More recent evidence has demonstrated that ILC2s are a source of IL-13 within the CNS. Indeed, IL-13 was found to be highly concentrated within the CSF of MS patients^81,82^. This finding is consistent with the large populations of ILC2s found in the CSF.

Although IL-13 has been shown to be largely protective in MS, studies involving its action in PD indicate a detrimental effect. In an experimental mouse model of PD, mice lacking IL-13Rα1 were protected against neuronal loss compared to their wild-type littermates ^83,84^, suggesting the neurotoxic effects of IL-13. Although one study demonstrated that neither IL-13 nor IL-4 induced cytotoxic effects on cultured dopaminergic neurons, both cytokines dose-dependently increased the toxicity of nontoxic doses of oxidants^85^. Therefore, the activation of IL-13Rα1 in PD may be one of the mechanisms by which dopaminergic neurons exhibit increased vulnerability to inflammation and ROS susceptibility.

### IL-10

Various cell types produce the immunoregulatory cytokine IL-10 as a response to neuroinflammatory cues. IL-10 was found to be expressed by astrocytes^86^ and microglial populations^87^. Although IL-10 has been extensively studied in astrocytes and microglia, the direct effect of ILC2-induced IL-10 on immune cell recruitment is limited. IL-10 downregulates pro-inflammatory cytokines, antigen presentation, and helper T-cell activation. Within the brain, IL-10 is locally synthesized and elevated during the course of most major CNS diseases to promote the survival of neurons and glial cells. Similar to peripheral IL-10, IL-10 within the brain blocks the effects of proapoptotic cytokines and promotes the expression of cell survival signals. For instance, IL-10 limits inflammation in the brain by (a) reducing the release of pro-inflammatory cytokines, (b) inhibiting receptor activation, and (c) suppressing cytokine receptor expression. Neural populations of ILC2s exhibit increases in IL-10 production after ischemia-reperfusion^88^. In fact, ILC-deficient mice have markedly reduced IL-10 levels associated with enhanced microglial reactivity and enhanced BBB damage. Meningeal engraftment of ILC2s increased IL-10 levels and ameliorated neuroinflammatory responses^89^. Collectively, this evidence demonstrates that ILC2-mediated IL-10 is a strong suppressor of neuroinflammation.

### TNFα

Within the periphery, lung populations of ILC2s were shown to selectively express TNFR2^90^. Interestingly, pharmacological blockade of TNFR2 reduced the population of ILC2s, which was associated with decreases in cytokine production and cell survival. This evidence is supplemented by similar observations from other groups, such as how the local elevation of TNFα activates ILC2s^91^. Within the brain, ILC2s were also modulated by TNFα. Intraperitoneal injection of IL-5, a potent cytokine released by activated ILC2s, led to a decrease in TNFα in aged mice^50^. Interestingly, AD has also been associated with increases in TNFα, and several anti-TNFα adjunct studies have demonstrated the amelioration of pathology^92,93^. It is possible that decreases in ILC2 populations within the meninges during aging and disease could potentially lead to an increase in TNFα. Correspondingly, if ILC2 populations could be increased, the detrimental expression of TNFα may be modulated. Future studies should determine whether ILC2s can ameliorate the damage induced by increases in TNFα.

### The CXCL16/CXCR6 axis

Although the previous sections of our review primarily discussed the effects of ILC2 modulation on cytokines specifically, chemokines have also been shown to interact with this unique cell type through cytokine modulation. Of interest, CXCL16 is commonly found within the brain and has been shown to exert neuroprotective effects against glutamatergic-induced excitotoxicity within neurons by modulating miniature excitatory synaptic currents (mEPSCs), particularly in the CA1 region of the hippocampus^94^. This modulation is vital to the survival and synaptic plasticity of neurons. Therefore, dysregulation of CXCL16 could lead to excitotoxicity and widespread neuronal injury, suggesting a possible mechanism of neurodegeneration observed in AD and PD. Apart from its effects on neurons, CXCL16 has also been shown to drive microglial polarization toward an anti-inflammatory phenotype upon stimulation with LPS^95^. Augmentation of this anti-inflammatory behavior may provide some degree of neuroprotection against neuroinflammatory diseases such as AD and PD.

The role of CXCL16 and its receptor, CXCR6, which form the “CXCL16/CXCR6-axis”, has been shown to be highly involved in the innate immune response in the periphery^96^. Indeed, CXCR6 is a common chemokine receptor expressed by ILC2s. CXCR6 deficiency in mice resulted in reductions in ILC2s and ILC1s/NK cells within the lung^97^. Chemotaxis assays demonstrated that CXCL16 directly induced the migration of ILC2s in mice. This effect was accompanied by the activation of classic neuroinflammatory markers such as ERK1/2, JNK, and NFκB. Although our understanding of ILC2 interactions with the CXCL16/CXCR6 axis in the brain is not as comprehensive as that in the periphery, these collective results show an extremely intimate relationship between ILC2s and the CXCL16/CXCR6 axis in the form of bidirectional modulation. Future investigations should elucidate the extent to which CXCL16 is modulated by ILC2s specifically within the brain and how these properties may change in response to the pathologies seen in various neurodegenerative diseases.

## Effect of ILC2s on Neurodegenerative Diseases

As seen from the lines of evidence discussed in previous sections, ILC2s are potent modulators of many downstream cytokines and chemokines. Although our understanding of ILC2s within the brain is in the early stages and far from complete, preliminary evidence suggests that this unique class of ILCs has previously underappreciated effects on the CNS. Experimentally, an increase in ILC2s through either chemical activation by IL-33 or direct ILC2 grafting within the mouse brain attenuates cognitive decline in aging mice^50^. Although this effect appears promising, the exact mechanisms by which this attenuation is modulated remain highly elusive. Many studies suggest that this observed cognitive improvement is due to the direct effects of cytokines and chemokines that modulate inflammation occurring due to neurological decline.

Although the alleviation of neuroinflammation occurs through cytokine modulation in this case, studies have demonstrated that cytokines and their receptors are difficult to therapeutically target in the context of disease because cytokine receptors are pleotropic in nature, and these receptors are not selectively expressed on specific neural cell types. For instance, IL-1β receptors are expressed on neurons, microglia, astrocytes, and oligodendrocytes^12–14,98^. Experimental mouse models revealed that IL-1β activation in astrocytes induced the p38 and NFκB pathways^99,100^. However, IL-1β activation in hippocampal neurons was shown to specifically activate p38 but not NFκB. Universal and nonspecific pharmacological targeting of IL-1β in this context will produce varying and, more importantly, unpredictable effects on neuroinflammation among different cell types. Direct pharmacological targeting of cytokines may in theory be an attractive process but remains a difficult challenge within the brain. For these reasons, ILC2s may be an attractive alternative therapeutic target. As a distinct brain-resident cell type, ILC2 upregulation can be easily targeted through different techniques, such as grafting or by secondary activation through cytokine stimulation. The sections below will discuss the value and potential of targeting ILC2s specifically in neurodegenerative diseases by examining some basic, animal, and preclinical evidence.

### Aging

Aging is recognized as a major risk factor for dementia-related disorders such as AD. This is unsurprising, as aging commonly also presents a chronic inflammatory state, as seen in neurodegenerative disorders. Similar to neurodegeneration, aging also results in the increased release of pro-inflammatory mediators (e.g., cytokines) such as IL-1, IL-6, and TNFα^101^, resulting in the upregulation of NFκB and affecting whole-body metabolism. At the cellular level, older adults tend to exhibit chronic inflammation from age-related cellular senescence associated with increased ROS and other cellular debris. Aberrant increases in macrophage infiltration into the brain from the periphery are also common observations^102^. In turn, increases in innate immune macrophages are also associated with increases in ILC2 responses^50^.

ILC2 development has been shown to be upregulated in the bone marrow of aged mice through increased notch signaling^44^. The average number of innate lymphoid progenitors was positively correlated with age. Similarly, ILC2s were also increased within the brain, particularly the choroid plexus, in aged mice^50^. Gene expression profiling of these mice revealed that there was upregulation of characteristic ILC2 genes (such as GATA3, IL-7R, and IL2Ra) in aged mice. Additionally, ILC2s in the aged brain are quiescent in homeostatic conditions but promptly proliferate upon activation by IL-33. Activated ILC2 populations are associated with improvements in cognition, as assessed by the Morris water maze and the object replacement test. These animal studies indicate that neural populations of ILC2s are attractive therapeutic targets in disease, as they have been demonstrated to be long-lived and can effectively switch between cycles of dormancy and proliferation. Despite these optimistic results, there is a problem with the route by which to target ILC2s in humans, which will be further addressed in section seven of this review.

### Alzheimer’s disease (AD)

Neuroinflammation is increasingly recognized as contributing to AD pathogenesis as much as senile plaques and tau neurofibrillary tangles^103^. Of interest, ILC2s have been shown to improve cognition through the upregulation of IL-5^50^. When ILC2s were treated with IL-5, there was an associated decrease in pro-inflammatory cytokines such as TNFα and CD68 in aged mice. Indeed, postmortem investigations of the AD brain have demonstrated increased TNFα levels across several regions^104^. Elevated TNFα levels in an AD mouse model enhanced Aβ production and decreased Aβ clearance^105^. Intracerebroventricular injection of infliximab, an anti-TNFα drug, reduced the TNFα load associated with decreases in Aβ plaques in an APP/PS1 mouse model^106^. Encouraging translational studies led to a small 6-month study in humans, and infliximab was introduced perispinally and led to improvements in the Mini-Mental State Examination (MMSE) scores of AD patients^107^. However, patients exhibited acclimatization to the drug dose, and perispinal drug accumulation was observed. Although TNFα may be an attractive target, direct modulation by drugs still presents many problems. As ILC2s show promising modulatory effects on TNFα, future studies should investigate whether it may be possible to modulate TNFα through pharmacological targeting of ILC2s.

Similarly, IL-33 has been shown to ameliorate synaptic impairment and memory deficits in APP/PS1 transgenic mice^55^, and impaired IL-33/ST2 signaling is commonly observed in AD patients^108^. Interestingly, ST2 is expressed on ILC2s and is regarded as an exclusive marker of this specific cell type. Furthermore, IL-33 has been shown to be a potent activator of ILC2s in several models of the lung, small intestine, spinal cord, and brain. ILC2 activation is associated with switching brain-resident microglia toward an anti-inflammatory phenotype, which is associated with reduced expression of IL-1β, IL-6, and NLRP3^55^. With regard to this study, it would be interesting to investigate whether the cognitive improvements observed here are mediated by the modulation of ILC2s.

To the best of our knowledge, there have been no direct investigations of ILC2-mediated modulation of tau hyperphosphorylation. Nonetheless, ILC2s demonstrate potent effects on many cytokines that have direct effects on tau hyperphosphorylation in both primary cell lines and mouse and preclinical models. Fung and colleagues demonstrated that the upregulation of IL-5 via ILC2 activation resulted in the attenuation of cognitive dysfunction^50^. However, the corresponding effect on tau pathology was unfortunately not investigated. Investigations into the neuroprotective effects of IL-5 demonstrated that treatment of PC-12 cell lines with IL-5 resulted in decreased Aβ_25-35_-induced tau hyperphosphorylation^109^. This effect was further associated with decreases in apoptotic signals through JAK2 signaling pathways. A similar phenomenon was observed in 3x Tg AD mice, in which IL-5 levels were significantly lowered compared to those of wild-type mice^110^. This evidence demonstrates that increased modulation of IL-5 may ameliorate AD pathology. Although a small AD patient cohort study showed that IL-5 (among TNFα and VEGF) was most strongly correlated with pathological severity^77^, few investigations have examined the effects of IL-5 as a preclinical target in humans. However, ILC2s share a strong modulatory relationship with IL-33 and IL-5. It is possible that these neuroprotective effects are modulated through ILC2s in the context of pathology. It is also likely that other ST2^+^ cells may target IL-33 and act in conjunction with ILC2s. Further studies should elucidate this phenomenon.

### Multiple sclerosis (MS)

MS is an autoimmune demyelinating disease characterized by myelin-specific T-cell infiltration through the BBB, which damages oligodendrocytes and nerve axons. Resident ILC2s are highly expressed in the meninges and proximally enclose the CSF^63^, serving as a critical gateway to neuroinflammation. Perhaps even more importantly, the recent discovery of lymphatic vessels within the CNS indicates a conduit for peripheral immune cells and macromolecules (e.g., free-flowing cytokines) to access the meninges and activate ILC2s.

To mirror the pathological effects observed in MS, rodent models of this disease, EAE, are often used. Consistent with other findings in the field, investigations into ILC2 behaviors in EAE demonstrate that these cells are resident within the meninges and are proximally juxtaposed to the BBB^111^. More interestingly, male *Kit*^W/Wv^ mice were found to have reduced ILC2 development and reduced population numbers within the spinal cord and brain compared to their wild-type counterparts^112,113^. Similarly, ILC2 populations in PLP_139–151_-immunized SJL females were found to be significantly lower than those in the controls^114^. As MS pathology is well known to be sexually dimorphic, the increases in ILC2s observed in both males and females provide one branch of unification in this otherwise complex disease.

Another significant observation within the field is that IL-33 administration to female mice prior to PLP_139–151_ immunization prevented EAE via ILC2 activation^114^. Male counterparts demonstrated increased IL-33 upon disease induction (due to testosterone-mediated activation of mast cells), and antibody blockade of IL-33 removed the initial protection against EAE in males. Indeed, reductions in IL-33 in disease models are highly associated with decreased ILC2 populations in PLP_139–151_-immunized mice, correlating with more severe disease phenotypes. This finding is unsurprising, as IL-33 is well known to be a potent activator of ILC2s both in the periphery and the CNS. Additionally, IL-33 has been shown to upregulate oligodendrocyte gene expression and myelination through p38/MAPK phosphorylation, thereby repairing myelination in damaged neurons^115^.

Despite these early optimistic results, other studies demonstrated that the protection offered by IL-33/ILC2 modulation is not consistently observed in all strains of mice^116^. It was hypothesized that this discrepancy is due to documented variations in serum testosterone levels in different strains. For instance, wild-type C57BL/6 male mice exhibit significantly lower testosterone levels, which are associated with decreased protection against EAE, than their SJL counterparts^117^. Clearly, the effects of mast cell populations and the corresponding cytokine responses can also contribute to this effect^114^. Future studies should investigate the intimate relationship between mast cells and ILC2 activation and whether their roles are interdependent in the context of neuroprotection in various EAE models.

There is currently substantial evidence that Th1-dominated immune responses in MS result in more severe phenotypes. Blood samples from untreated patients diagnosed with the clinically isolated syndrome (CIS), relapsing-remitting MS (RRMS) (which are associated with more severe pathologies), or progressive MS (which is associated with less severe pathologies) were tested for plasma levels of different cytokines^118^. The results demonstrated that CIS and RRMS patients had higher levels of Th1 cells, which was associated with the activation of IFNγ, whereas the less severe pathologies seen in progressive MS suggested Th2 expression, such as those seen in ILC2s. Shifting the pathogenic Th response in MS and EAE models from Th1 to Th2 seems to be a viable therapeutic strategy. Indeed, FDA-approved drugs such as glatiramer acetate and dimethyl fumarate induce this effect; however, their application comes with side effects^119,120^. In view of these reasons, using ILC2s or mast cells to promote a strong Th2 response could prove useful. Future investigations should examine whether IL-33 can be directly administered to upregulate ILC2 activity and whether this strategy may be effective in ameliorating various MS/EAE symptoms without inducing many adverse effects.

### Parkinson’s disease (PD)

PD can be described as a neuroinflammatory disease characterized by the progressive loss of dopaminergic neurons within the SNc and the striatum, accompanied by the aggregation of alpha-synuclein and Lewy body inclusions^121^. Patients commonly exhibit bradykinesia, resting tremors, and muscle rigidity, as well as cognitive decline in later stages of the disease. Of interest, clinical studies of disease etiology revealed that systemic inflammatory diseases such as irritable bowel syndrome (IBS) are highly correlated with PD^122,123^. This connection is well understood largely due to a decade of research investigating the intimate link between the gut-brain axis and how microbiota distributions can affect both the neuroendocrine and nervous systems.

ILC2s are located proximal to neurons in the intestine and share intercellular contacts. For instance, ILC2s colocalize closely with adrenergic neurons located in the villi, parenchyma, and mesenteric lymph nodes^124,125^. Additionally, ILC2s are also juxtaposed with cholinergic neurons in the lamina propria of the small intestine^125^. The close proximity of nerve fibers suggests that ILC2s are regulated by the nervous system through multiple gateways in the intestine. Of interest, β2 adrenergic receptor (β_2_AR) signaling was shown to impair ILC2 responses in the intestine^126^. In particular, the proliferation of ILC2s was suppressed by β_2_AR stimulation. β_2_AR signaling is also linked to the upregulated transcription of α-synuclein in PD, as shown in cells and genetic sequencing of tissues derived from human PD patients^127^.

**Fig. 4:**
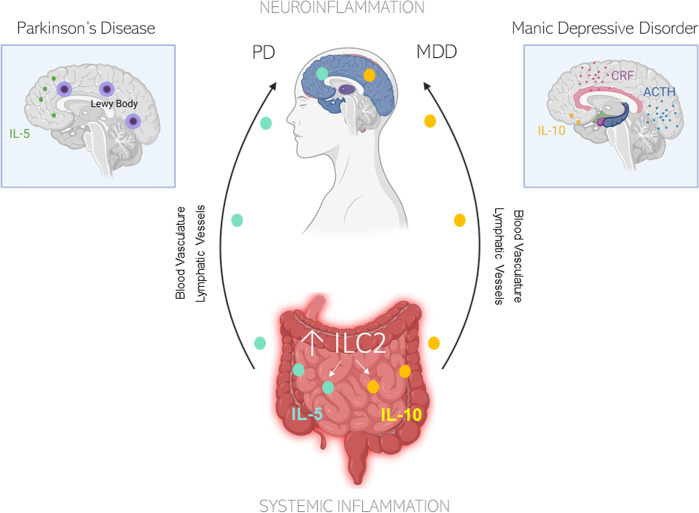
Schematic diagram illustrating the possible role of ILC2s in modulating the gut-brain axis. For example, IL-5 and IL-10 are readily induced by ILC2s in IBS. Released cytokines in the intestines can travel from peripheral systems into the brain through either the blood vasculature or lymphatic vessels or through secondary activation from roaming macrophage populations. In Parkinson’s disease and IBS, serum levels of IL-5 are increased^155^,^128^. In IBS, IL-10 levels are increased. Similarly, IL-10 levels have been shown to induce CRF and ACTH release^145^. It is likely that the ILC2 release of downstream cytokines can highly modulate both systemic inflammation and neuroinflammation, thereby explaining a possible route for gut-brain communication. Illustration created in part with *BioRender.com*.

Moreover, disruptions in circadian rhythms have been linked to PD. When intestinal ILC2 populations are incubated with vasoactive intestinal peptides (VIPs), a surge in IL-5 occurs.^128^ Animal studies have demonstrated that VIPs stimulate ILC2-dependent production of IL-5 in response to circadian cues^129^. It is unsurprising that the dysregulation of circadian cues can change the gut microbiota and predispose individuals to pathological burdens^130^. Additionally, ILC2s have been shown to be the predominant cell type that regulates IL-10 expression in the intestine^131^. IBS patients exhibit significantly lower IL-10 levels than age-matched controls, and a lack of IL-10 compromises the restoration of the small intestine epithelial barrier by MHCII^+^ cells (which is also expressed on ILC2s) after NSAID-induced injury^132^. Given the intimate neuroendocrinological link between the intestine and the nervous system, it would be unsurprising if this peripheral CNS communication is modulated in part by ILC2 (Fig. 4).

Apart from IL-5, an important downstream cytokine activated by ILC2s is IL-13. The human *Il13ra1* gene expressed on the X-chromosome and the PARK12 gene are highly implicated in PD susceptibility^133^. Various studies have demonstrated that chemical or genetic elimination of IL-4/IL-13 ameliorates both cognitive and motor symptoms of PD. Interestingly, in a study performed by Fung and colleagues, cognitive deficits induced by aging were ameliorated by the injection of IL-5 but not IL-13. Interestingly, IL-13 deficiencies in AD mouse models significantly impaired working memory^50^. It seems that the role of IL-13, even within different neurodegenerative diseases, induces different effects, and the reasons remain elusive. Based on this evidence, ILC2 modulation in the context of PD may be far more complex than initially thought due to its pleotropic nature. Indeed, ILC2 activation induces both IL-5 and IL-13 downstream with very little specificity. Overall, the role of ILC2s and their downstream cytokine effects on PD, especially with regard to IL-13, warrants closer scrutiny in comparison with other disease models.

### Depressive disorder (MDD)

Clinical diagnosis of MDD herein referred to as depression, mainly occurs through examinations of symptoms of despondency, decreased activity, and anhedonia in patients. Often, a depression diagnosis is difficult to make, as clinical depression can manifest in individuals in many ways. However, studies on the neurophysiology of patients with depression revealed consistent decreases in dopaminergic, serotonergic, and noradrenergic transmission within the brain, which is known as the monoamine hypothesis^134^. The augmentation of monoamine neurotransmission by anthocyanin and upregulation of BDNF expression exhibit ameliorative effects on depression in various mouse models via the promotion of neurogenesis^135^. However, most currently used antidepression treatments that attempt to upregulate monoamine transmission or reuptake have shown limited efficacy. For instance, there is a latency in the response to antidepressants in many patients, while some patients even demonstrate refractory behavior to antidepressants available in the market^134,136^. Therefore, monoamine transmission may not be the only pathophysiological mechanism driving depression, and monoamine-alternative treatments or targets must be further investigated.

Of interest, a diverse range of evidence has suggested the involvement of the innate immune system in MDD pathology. For a decade, it has been well established that depressive patients exhibit increased circulating levels of monocytes and other inflammatory markers (e.g., pro-inflammatory cytokines, chemokines, prostaglandins)^137^. This finding is indicative of immune recruitment as a response to the neurobiological changes associated with MDD. Mice exposed to social defeat stress show monocyte infiltration in brain regions associated with depression and anxiety^138^. In fact, pharmacological downregulation of Ly6C(hi) monocytes within the periphery results in the amelioration of depressive behaviors^139^. Early studies of adaptive T-cell alterations in depression showed that antidepressant treatments associated with the amelioration of depression behavior restored Th2 imbalances to an extent.

As ILC2s are Th2-type cells, it comes as no surprise that recent studies investigating links between inflammatory bowel disease (IBD) and depression suggest that ILC2s modulate this connection. Investigations such as these are extremely relevant, as the comorbidity of depressive symptoms with IBD, or vice versa, are correlated with poorer clinical outcomes^140,141^. As previously demonstrated, ILC2s are closely associated with β_2_-noradrenergic neurons in the human colonic mucosa and epithelium^142^, as well as the meninges in the brain^49^. Due to the gut-brain axis, it would be unsurprising if the modulation of ß_2_-adrenergic neurons in the colon can be affected by resident ILC2 populations, which further elicit microglial activation and secondary cytokine activation in the brain.

To date, there have been no direct investigations on neural populations of ILC2s and their effects on the neurophysiology of depression. However, investigations on ILC1/NK cell populations demonstrated that the upregulation of IL-12 promotes the expression of PD-1 on the surface of NK cells in a model of hypothalamic pituitary adrenal (HPA) axis infection^143^. Overactivation and inflammation of the HPA axis are heavily implicated in MDD. Despite these early results, it is clear that ILC2s are the main population of ILCs within the brain. As of now, we are still unsure whether ILC2s can modulate the HPA. However, studies have shown that immune activation by LPS can induce inflammation in the meninges, which later occurs in the hypothalamus^144^. In line with this concept, IL-10 enhances the release of corticotropin-releasing factor (CRF) and corticotropin (ACTH) in hypothalamic and pituitary tissues, respectively^145^. Furthermore, endogenous IL-10 can contribute to glucocorticosteroid production following further exposure to stressors associated with inflammation. Overall, IL-10 is vital to the modulation of inflammation, and its selective modulation by ILC2s in the intestine suggest a possible link between neuroendocrine activities in human physiology. Further studies should investigate whether ILC2s in fact modulate the microbiota associated with neurodegenerative disease and regulate neuroendocrinological connections to a larger extent than previously appreciated.

### Challenges in the specific targeting of ILC2s

As seen from the evidence described previously, ILC2s in the choroid plexus and meninges can modulate many downstream factors in the brain via cytokine release. Although our understanding of ILC2 populations in the brain is still early and far from complete, initial evidence from the last 5 years does demonstrate more robust cognitive modulation by ILC2s than other ILC types. Although ILC1s and ILC3s may influence targets that have indirect effects on the CNS^146,147^, ILC2s are most heavily implicated in directly modulating brain cognition in neurological diseases. Several groups have directly demonstrated that ILC2 activity in the brain is downregulated in many instances of neurodegenerative damage (e.g., aging, AD, MS, SCI)^49,50,64^ and that ILC2 activation via IL-33 supplementation can attenuate cognitive decline^55^. Collectively, this evidence demonstrates that ILC2s may serve as attractive molecular targets, as they have also been demonstrated to be long-lived and effectively switch between cycles of dormancy and proliferation.

In the CNS, ILC2s were found in the choroid plexus and meninges. In an early study, ILC2s in the meninges were found to be CD45^+^, Thy1.2^+^, and ST2^+^ cells that express C-kit, Sca1, IL-7Rα, and CD25 on their cell surface^49^. ILC2 populations in the choroid plexus were found to express similar characteristic genes, such as GATA3^50^. These populations have also been shown to be potently activated by IL-33 and secrete IL-5 and IL-13 readily upon stimulation within the brain. Individually, IL-5 and IL-13 have been shown to modulate neuroinflammation in neurodegenerative diseases. For instance, IL-5 has been shown to positively modulate AD pathology via decreased tau hyperphosphorylation^109^. IL-13 also demonstrates positive effects on AD by decreasing the production of the pro-inflammatory cytokine IL-6 in microglia specifically exposed to oligomeric Aβ_(1–42)_^148,149^. Interestingly, other studies suggest that IL-13 induces oxidative stress in hippocampal neurons specifically in response to Aβ_(1–42)_^150^. It seems that IL-13 may elicit differential effects on the pathological outcomes depending on the cell type targeted. As previously mentioned, targeting downstream cytokines is an ongoing difficulty, as their receptors are so diversely distributed on many cell types, even though they have different and cell-specific modulatory effects. In this respect, targeting ILC2s will theoretically promote nonselective upregulation of both IL-5 and IL-13, regardless of whether these cytokines can induce neuroprotection. Interestingly, a study investigating the effects of aging on ILC2 levels in the choroid plexus indicated that ILC2s selectively stimulated IL-5, with comparatively few modulatory effects due to IL-13. Indeed, the stimulation of IL-5 via ILC2s reduced the production of pro-inflammatory cytokines such as TNFα. Notably, IL-5 produced in the choroid plexus induces more direct effects on microglia and neurons than IL-13 produced in the meninges. Future investigations examining the neuroprotective effects of ILC2s should specifically characterize the extent to which IL-5 and IL-13 are activated and modulate disease.

Although ILC2s and their downstream effectors may modulate several aspects of neurological disease, it remains unclear how ILC2s can be specifically targeted without affecting other ILC subtypes (i.e., NK cells/ILC1s or ILC3s). Experimentally, early studies attempted to isolate CNS populations of ILC2s through the close observation of specific expression markers on the cell surface. For example, Gadani and colleagues isolated ILC2s in the spinal cord region by first using a global ILC depletion strategy with Rag^−/−^ animals that lacked T and B cells but maintained Thy1.2 expression, which is commonly found specifically on ILC2s^49^. Specific silencing of ILC2s was attempted through anti-Thy1.2 depletion but was found to be ineffective in meningeal populations of ILC2s.

There is a certain complexity with targeted KO of ILC2s using traditional methods based on surface marker expression. In an alternative attempt, lung-derived ILC2s were adoptively reintroduced into the cisterna magna of ST2^−/−^−/− mice prior to inducing a spinal cord injection model. Although lung ILC2 populations were used for the adoptive transfer experiments, they were selected based on prior screening for neuroprotective gene expression. These populations of lung ILC2s may have neuroprotective profiles that differ from meningeal ILC2s and therefore may not reflect the full potential of neural populations. In fact, due to the relatively sparse distribution of ILC2s in the meninges of the spinal cord, more meningeal ILC2s may be needed to achieve the same spinal protective efficacy demonstrated by the adoptive transfer method.

More recent investigations into populations of ILC2s in the choroid plexus stepped away from utilizing lung extractions of ILC2s and instead used lentiviral transduction of ILC2/b6 cell lines in aged mice. The ILC2/b6 cell line is an immortalized ILC2 cell line that exhibits similar molecular and functional characteristics as activated ILC2s. Investigations showed that this cell line expresses both Bcl11b and Id2, while early T-lineage cells produce only Bcl11b^151,152^. Indeed, Bcl11b occupies distinct sites in lineage-specific patterns of distinctive target genes, indicating that ILC2s and early T-lineage cells are in fact transcriptionally different. Consistent with our knowledge, this ILC2 line responds to IL-33 stimulation and produces IL-4 and IL-13 in vitro. More specifically, these cells also lack the production of IL-22, IL-17, and IFNγ, which are typically modulated by other ILC types^153^. Although this ILC2/b6 cell line was demonstrated to have the modulatory characteristics of resident CNS populations of ILC2s, future investigations should attempt to target these ILC2 populations directly, as there are substantial differences between the behaviors of these cell types once they have been isolated and grown in an artificial environment before introduction into animals.

## Concluding Remarks

Studies on the role of ILC2s within the CNS are novel and timely, given the recent discovery of the immune axis in brain lymphatics. Early exploration of the topic demonstrates the potential for ILC2s to modulate neurodegeneration and shows their promise. However, there are still evident gaps in the mechanistic understanding of how ILC2 populations specifically act and respond to damage within the CNS. This gap may be due in part to a lack of technical tools to directly isolate and manipulate brain-resident populations of ILC2s at this stage. Upon availability of these tools, investigations should examine the effect of brain-resident populations on long-lived toxic proteins such as tau or TDP-43, as these factors are often present in various neurodegenerative disorders^154^. Currently, ILC2 behavior has been studied in the context of normal aging and consequent cognitive decline. However, neither the effect of these cells on specific pathologies (for instance, aging-associated tau aggregations) nor their distribution in specific neural structures (e.g., hippocampus or prefrontal cortex) have been discussed. These topics could be interesting areas for further validation and would prove most useful in determining the therapeutic potential of ILC2s.

Given the available literature, ILC2s can also serve as an attractive link between systemic inflammation and neuroinflammation. Shared monoaminergic connections between the brain and small intestine/colon have been shown to be modulated by ILC2s^126^. However, little information exists on the putative effects of ILC2s on other peripheral organs as a result of primary manipulations of their neural population. It is important to investigate whether the manipulation of neural populations of ILC2s can activate peripheral populations and affect homeostatic cell behaviors in organs such as the lung and GI tract. Future efforts should attempt to understand the genetic or transcriptional similarities between ILC2s within the CNS and the periphery, as specific targeting of brain ILC2s and downstream cytokines is vital if we are to manipulate this cell type in the context of disease. In summary, ILC2s and their downstream effectors may be effective targets in the CNS. However, many challenges remain regarding the identification, experimental targeting, and characterization of ILC2s in brain health and disease.
